# Author Correction: High-resolution sampling of beam-driven plasma wakefields

**DOI:** 10.1038/s41467-020-20676-1

**Published:** 2021-01-08

**Authors:** S. Schröder, C. A. Lindstrøm, S. Bohlen, G. Boyle, R. D’Arcy, S. Diederichs, M. J. Garland, P. Gonzalez, A. Knetsch, V. Libov, P. Niknejadi, Kris Põder, L. Schaper, B. Schmidt, B. Sheeran, G. Tauscher, S. Wesch, J. Zemella, M. Zeng, J. Osterhoff

**Affiliations:** 1 Deutsches Elektronen-Synchrotron DESY, Notkestraße 85, 22607 Hamburg, Germany; 2 Universität Hamburg, Mittelweg 177, 20148 Hamburg, Germany

**Keywords:** Plasma-based accelerators, Characterization and analytical techniques

Correction to: *Nature Communications* 10.1038/s41467-020-19811-9, published online 25 November 2020.

The original version of this Article contained an error in the spelling of the author Kris Põder, which was incorrectly given as K. Pøder.

This has now been corrected in both the PDF and HTML versions of the Article.

