# Supplementary material for: High-resolution sampling of beam-driven plasma wakefields
Source: Nat Commun. 2020 Nov 25;11:5984. doi: 10.1038/s41467-020-19811-9 (PMC7689520; doi:10.1038/s41467-020-19811-9)
Supplement: Supplementary file 1 — Supplementary Information [file 41467_2020_19811_MOESM1_ESM.pdf]

Supplementary Information

High-resolution sampling of beam-driven plasma wakefields

S. Schröder[1,2,*], C.A. Lindstrøm[1], S. Bohlen[1,2], G. Boyle[1], R. D'Arcy[1], S. Diederichs[1,2], M.J. Garland[1], P. Gonzalez[1,2], A. Knetsch[1], V. Libov[1], P. Niknejadi[1], K. Põder[1], L. Schaper[1], B. Schmidt[1], B. Sheeran[1,2], G. Tauscher[1,2], S. Wesch[1], J. Zemella[1], M. Zeng[1], and J. Osterhoff[1]

[1]Deutsches Elektronen-Synchrotron DESY, Notkestraße 85, 22607 Hamburg, Germany

[2]Universität Hamburg, Mittelweg 177, 20148 Hamburg, Germany

[*]*email: sarah.schroeder@desy.de*

Supplementary Figure 1 **2**

Supplementary Figure 2 **3**

Supplementary References **4**

Supplementary Figure 1

Temporal calibration of tail-collimator from the measured longitudinal-phase-space distribution. **a**, A virtual tail-collimator scan is performed on the reconstructed longitudinal phase space to identify the longitudinal position of each energy slice. **b**, The remaining (uncollimated) charge at each step of the actual tail-collimator scan (Fig. 3) is compared to the cumulative charge below each energy in the virtual tail-collimator scan. **c**, This allows the longitudinal slice position of each tail-collimator step to be determined.

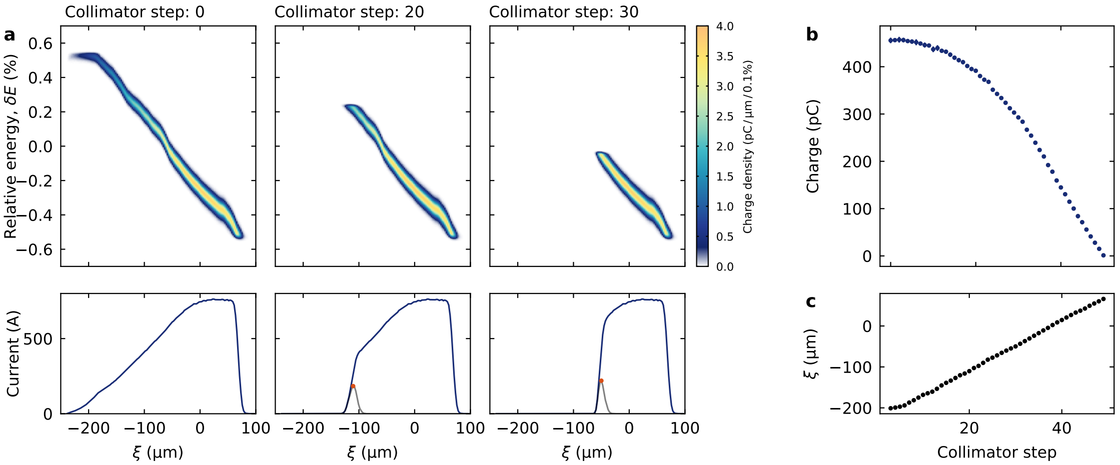

Supplementary Figure 2

Modelling the plasma density profile. a, Longitudinally resolved on-axis plasma density (black error bars) in a 95–5% argon–hydrogen mixture (percentage by number density) measured for times between 4 and 10 µs. The coloured surface represents a fitted function of the form

$$\mathrm{n}(z,t) = \frac{1}{2}\mathrm{A}(t)\left[\mathrm{erf}\left(\frac{z-c_1}{\sqrt{4c_3(t+c_4)}}\right) - \mathrm{erf}\left(\frac{z-c_2}{\sqrt{4c_3(t+c_4)}}\right)\right],$$

where z is the longitudinal position, t is the trigger delay timing, and the constants are $c_1 = 14.1$ mm, $c_2 = 18.4$ mm, $c_3 = 2.97\ \mathrm{mm}^2\,\mu\mathrm{s}^{-1}$, $c_4 = 4.16$ µs. $\mathrm{A}(t)$ represents a scaling factor that ensures that each measurement set has the appropriate average density. This functional form has been chosen because of its close association with the one-dimensional diffusion equation for an initially uniform source.[1] **b,** The fitted surface is linearly interpolated to the discharge trigger delay timings of the measurements 7.5 µs / 9.3 µs and scaled to an average density of $4.4 \times 10^{16}\ \mathrm{cm}^{-3}$ (blue curve) / $2.4 \times 10^{16}\ \mathrm{cm}^{-3}$ (grey curve) respectively.

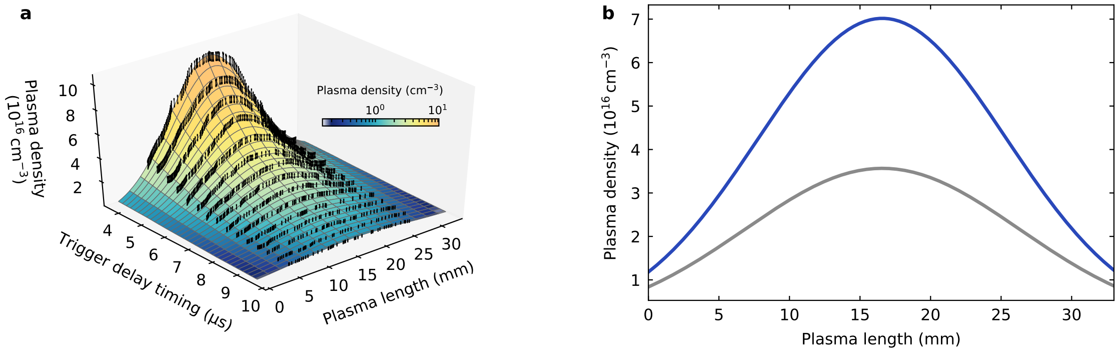

Supplementary References

[1] Morse, P. M. C. & Feshbach, H. *Methods of theoretical physics*. International Series in Pure and Applied Physics (McGraw-Hill, 1953).
